# Physicochemical understanding of biomineralization by molecular vibrational spectroscopy: From mechanism to nature

**DOI:** 10.1002/EXP.20230033

**Published:** 2023-07-26

**Authors:** Hao Liu, Hui Jiang, Xiaohui Liu, Xuemei Wang

**Affiliations:** ^1^ State Key Laboratory of Digital Medical Engineering School of Biological Science and Medical Engineering Southeast University Nanjing Jiangsu China

**Keywords:** analytical chemistry, biomineralization, calcification, crystallization, Raman spectroscopy

## Abstract

The process and mechanism of biomineralization and relevant physicochemical properties of mineral crystals are remarkably sophisticated multidisciplinary fields that include biology, chemistry, physics, and materials science. The components of the organic matter, structural construction of minerals, and related mechanical interaction, etc., could help to reveal the unique nature of the special mineralization process. Herein, the paper provides an overview of the biomineralization process from the perspective of molecular vibrational spectroscopy, including the physicochemical properties of biomineralized tissues, from physiological to applied mineralization. These physicochemical characteristics closely to the hierarchical mineralization process include biological crystal defects, chemical bonding, atomic doping, structural changes, and content changes in organic matter, along with the interface between biocrystals and organic matter as well as the specific mechanical effects for hardness and toughness. Based on those observations, the special physiological properties of mineralization for enamel and bone, as well as the possible mechanism of pathological mineralization and calcification such as atherosclerosis, tumor micro mineralization, and urolithiasis are also reviewed and discussed. Indeed, the clearly defined physicochemical properties of mineral crystals could pave the way for studies on the mechanisms and applications.

## Graphical abstract

An overview of the biomineralization process from the perspective of molecular vibrational spectroscopy, including the physicochemical properties of biomineralized tissues, from physiological to applied mineralization. Indeed, the clearly defined physicochemical properties of mineral crystals could pave the way for studies on the mechanisms and applications.

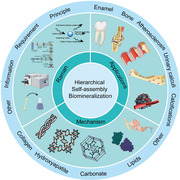

## 1 INTRODUCTION

The main mineral components of the organism are produced by biomineralization, which is an important factor affecting the development of bone and teeth mineralization in the organism.^[^ ^1^, ^2^, ^3^, ^4^ ^]^ In addition, biomineralization is closely related to cardiovascular diseases, urological diseases, and liver and kidney stones.^[^ ^5^, ^6^, ^7^, ^8^ ^]^ The biomineralization process of teeth, bones, and pathological stones is outlined as intracellular mineralization and extracellular mineralization, a process of crystallization of hydroxyapatite and calcium carbonate under the regulation of organic matter.^[^ ^9^, ^10^, ^11^, ^12^ ^]^ At the molecular level, it is a kind of ordered hierarchical regulation and assembly of macromolecules, which leads to the crystallization and growth of the inorganic mineral phase.^[^ ^13^, ^14^, ^15^ ^]^ The organic phase assembles, and the inorganic phase nucleates, crystallizes, and grows.^[^ ^16^, ^17^, ^18^ ^]^ Self‐assembly of insoluble organic matter in specific cellular or tissue regions constructs the mineralized microenvironment, which includes organic macromolecular structure, orientation, and mineralization sites.^[^ ^19^, ^20^, ^21^ ^]^ The ions required for mineralization are nucleated at specific sites in the microenvironment by weak interactions such as hydrogen bonding, electrostatic forces, and van der Waals forces.^[^ ^1^, ^4^, ^22^, ^23^ ^]^ During the growth of the inorganic phase, the size, morphology, orientation, and structure of the crystals are regulated by the organic matter of the organism.^[^ ^24^, ^25^, ^26^ ^]^ The inorganic phase acquires a multi‐level assembly structure by graded regulation.^[^ ^27^ ^]^

Physiological mineralization is one of the most important biomineralization processes in biological development.^[^ ^28^, ^29^, ^30^ ^]^ Mineralization processes such as enamel, dentin, bone, and cartilage mineralization are typical hierarchical self‐assembly mineralization among others.^[^ ^28^, ^29^, ^31^ ^]^ The enamel organ is formed by odontoblasts and ameloblasts at specific developmental periods and is subdivided into the bud, cap, and terminal phases.^[^ ^32^, ^33^, ^34^ ^]^ Thus the enamel and dentin mineralization involves close interaction with odontoblasts and ameloblasts. Among the signaling pathways, wingless (Wnt), DNA mismatch repair homeobox 1/2 (Msx1/2), bone morphogenetic protein (Bmp), and runt‐related transcription factors 2 (Runx2), and disheveled homologous 2 (Dlx2) are involved in the mineralization process of enamel and dentin.^[^ ^2^, ^34^, ^35^, ^36^, ^37^ ^]^ These signaling proteins regulate the expression and secretion of ameloblastin (AMBN), amelogenin (AMELX), enamelin (ENAM), alkaline phosphatase, matrix metalloproteinase 20 (MMP20) (ALP), and kallikrein‐related peptidase 4 (KLK4).^[^ ^38^, ^39^, ^40^, ^41^, ^42^ ^]^ This process occurs after the tenth week of embryonic in humans and after the 13th day of embryonic in mice. Apoptosis of enamel‐forming cells at a later stage of development results in the failure of enamel regeneration, which is different from bone mineralization.^[^ ^22^, ^43^, ^44^ ^]^ Bone formation also begins at the embryonic stage, during which mesenchymal cells aggregate to form a cohesive mass that approximates the shape and location of the bone.^[^ ^45^, ^46^, ^47^ ^]^ Then, they differentiate into osteoblasts, osteoclasts, and osteocytes, which eventually participate in bone mineralization.^[^ ^48^, ^49^ ^]^ Osteoblasts induce to become terminally differentiated cells that produce extracellular matrix proteins such as collagen and promote bone mineralization.^[^ ^50^, ^51^ ^]^ Osteoclasts have the opposite function of maintaining the dynamic balance of bone mineralization. The biological understanding of this process has been well‐reviewed and summarized.^[^ ^2^, ^50^, ^52^, ^53^, ^54^ ^]^ In contrast, the physicochemical properties of the inorganic and organic phases during mineralization have not been further reviewed and understood, although some research progress has been made recently.

Pathological mineralization is different in vivo for the hierarchical self‐assembly mineralization process. The simplest example is the occurrence of calculus, which is caused by bacteria on the enamel surface forming a biofilm that further induces the onset of mineralization.^[^ ^55^, ^56^, ^57^, ^58^, ^59^ ^]^ The end‐stage of atherosclerosis is pathological mineralization, which generally begins with lipid and complex sugar accumulation, hemorrhage, and thrombosis, followed by fibrous tissue proliferation and mineral crystallization.^[^ ^7^, ^60^, ^61^, ^62^ ^]^ Urinary stones are a complex of calcium oxalate and carbonate apatite, which is also due to pathological mineralization.^[^ ^8^, ^63^, ^64^ ^]^ Or mineralized nodules that occur in malignant tissues, such as breast cancer.^[^ ^65^, ^66^, ^67^, ^68^ ^]^ Interestingly, some studies have used this type of mineralization to induce intracellular mineralization in tumor cells for tumor therapy or fluorescence detection.^[^ ^69^, ^70^, ^71^ ^]^ The gold nanoparticles or nanoclusters produced by this intracellular mineralization may be nucleic acid‐induced for their nucleation.^[^ ^72^, ^73^, ^74^, ^75^ ^]^ A better understanding of the mechanisms underlying pathological mineralization and how biomolecules regulate the physicochemical properties of the inorganic phase is crucial.

**SCHEME 1:**
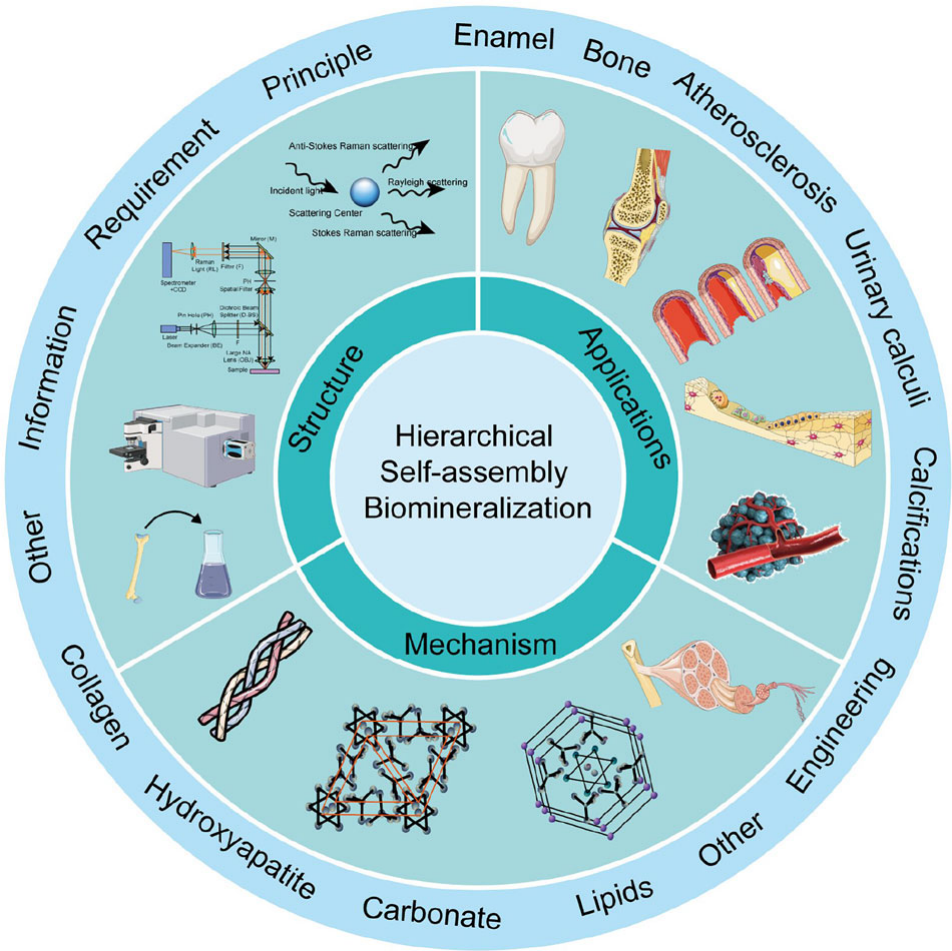
Schematic illustration of relevant nano‐structures, mechanisms, and applications of biomineralization.

**FIGURE 1:**
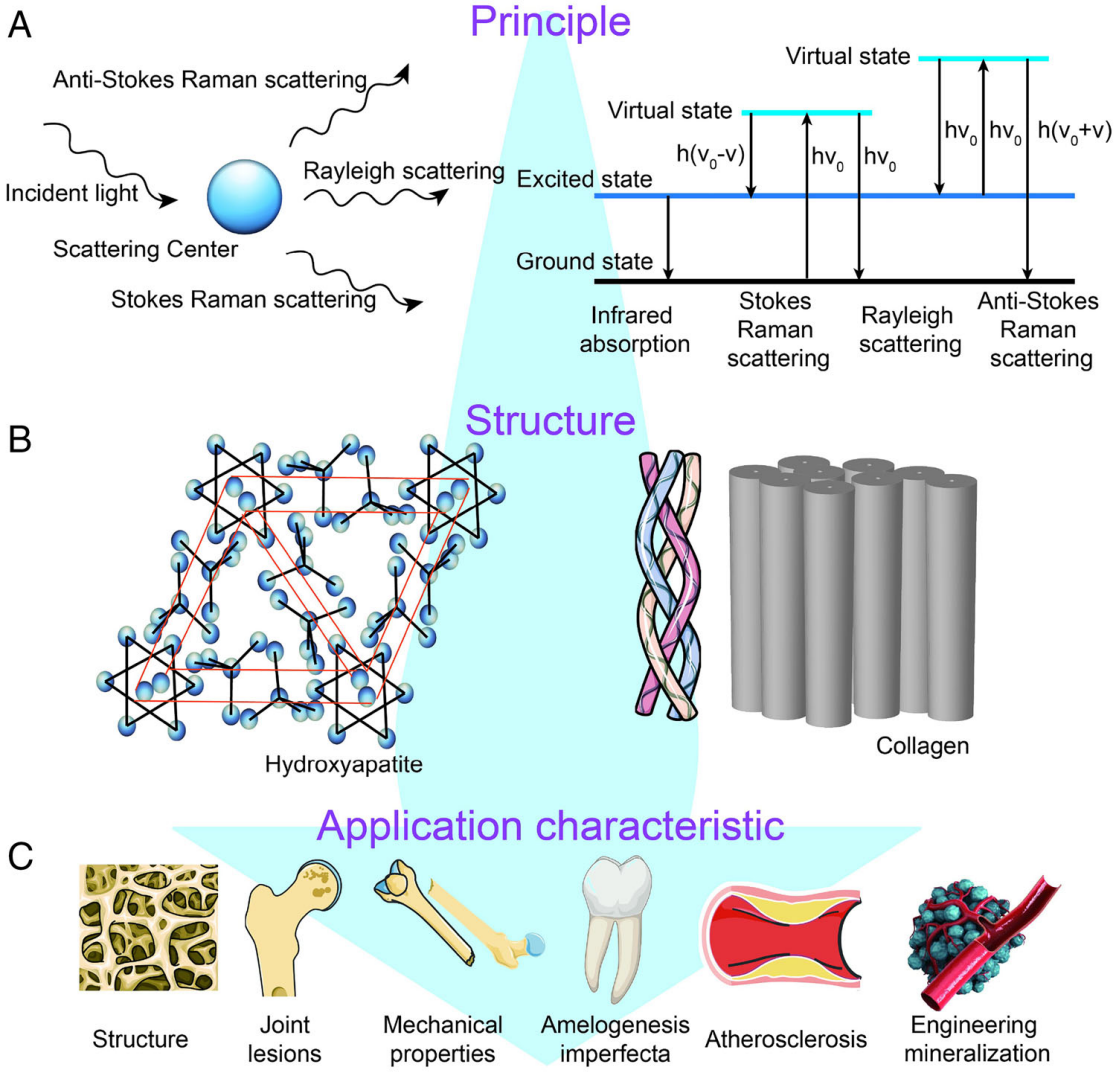
Schematic diagram of structural analysis process based on Raman spectroscopy. A, Raman spectra principle. B, Typical structure of biomineralization. C, Multiple structural forms of biomineralization.

Therefore, in this review, we have reviewed the hierarchical self‐assembly mineralization process and discussed the specific changes in physicochemical properties during physiological and pathological mineralization summarized in terms of molecular vibrational spectroscopy (Scheme 1). The important role of Raman spectroscopy in revealing the physicochemical properties of biomineralization is elucidated and the relevant experimental conditions of Raman spectroscopy for illustrating tissues or microenvironments that induce biomineralization in vivo and in vitro experiments are also exploited (Figure 1A). From these studies, it is possible to reveal the dynamic structural changes of inorganic and organic phases during the special process of biomineralization, including chemical bonding, crystal size, crystal orientation, organic component content, etc. (Figure 1B). And the advance of recent research progress of Raman spectroscopy for illustrating the unique biomineralization process is evaluated and discussed (Figure 1C). This review article aims to explore the nature of the hierarchical self‐assembly mineralization process and provide fundamental evidence for an understanding of why and how the specific alteration of physicochemical properties occurs during the related biomineralization process. The hierarchical self‐assembly mineralization process could significantly contribute to the formulation of new biomineralization nano‐structures, which offers new strategies for realizing the early theranostics of some difficult diseases like heart diseases and cancers.

## 2 PHYSICOCHEMICAL CHARACTERISTICS OF BIOMINERALIZATION

### 2.1 Principle of Raman spectroscopy

Vibrational spectroscopy such as Raman and infrared (IR) spectroscopy are usually used as high‐sensitive analytical tools for biomedical research.^[^ ^76^, ^77^, ^78^, ^79^ ^]^ Raman spectroscopy is widely used in biomineralization because of its high resolution, accuracy and low costs.^[^ ^80^, ^81^, ^82^, ^83^ ^]^ The spectral features of Raman spectroscopy are obtained by different interaction modes between the incident radiation and the sample, which is the inelastic scattering of the incident light caused by changes in the polarizability of the target molecules (Figure 1A). It is a process in which the energy of the scattered photon is different from the energy of the incident photon and is described as the Raman effect.^[^ ^84^, ^85^ ^]^ This inelastic process puts the molecule in a vibrational state. Scattering in which the energy of the scattered photon is equal to the energy of the incident photon is often referred to as Rayleigh scattering.^[^ ^86^ ^]^ When the energy of the scattered photon is lower than the energy of the incident photon, it is called Stokes–Raman scattering, and the opposite phenomenon is called anti‐Stokes–Raman scattering (Figure 1A). The Stokes–Raman signal of the molecule is stronger than the anti‐Stokes signal because the overall energy state is controlled by thermal statistics.^[^ ^87^, ^88^, ^89^, ^90^ ^]^ Therefore, Stokes–Raman scattering is now used to analyze minerals, phospholipids, collagen, and others in biomineralized tissues.^[^ ^91^, ^92^, ^93^ ^]^

### 2.2 The advance of Raman spectroscopic study on biomineralization

**FIGURE 2:**
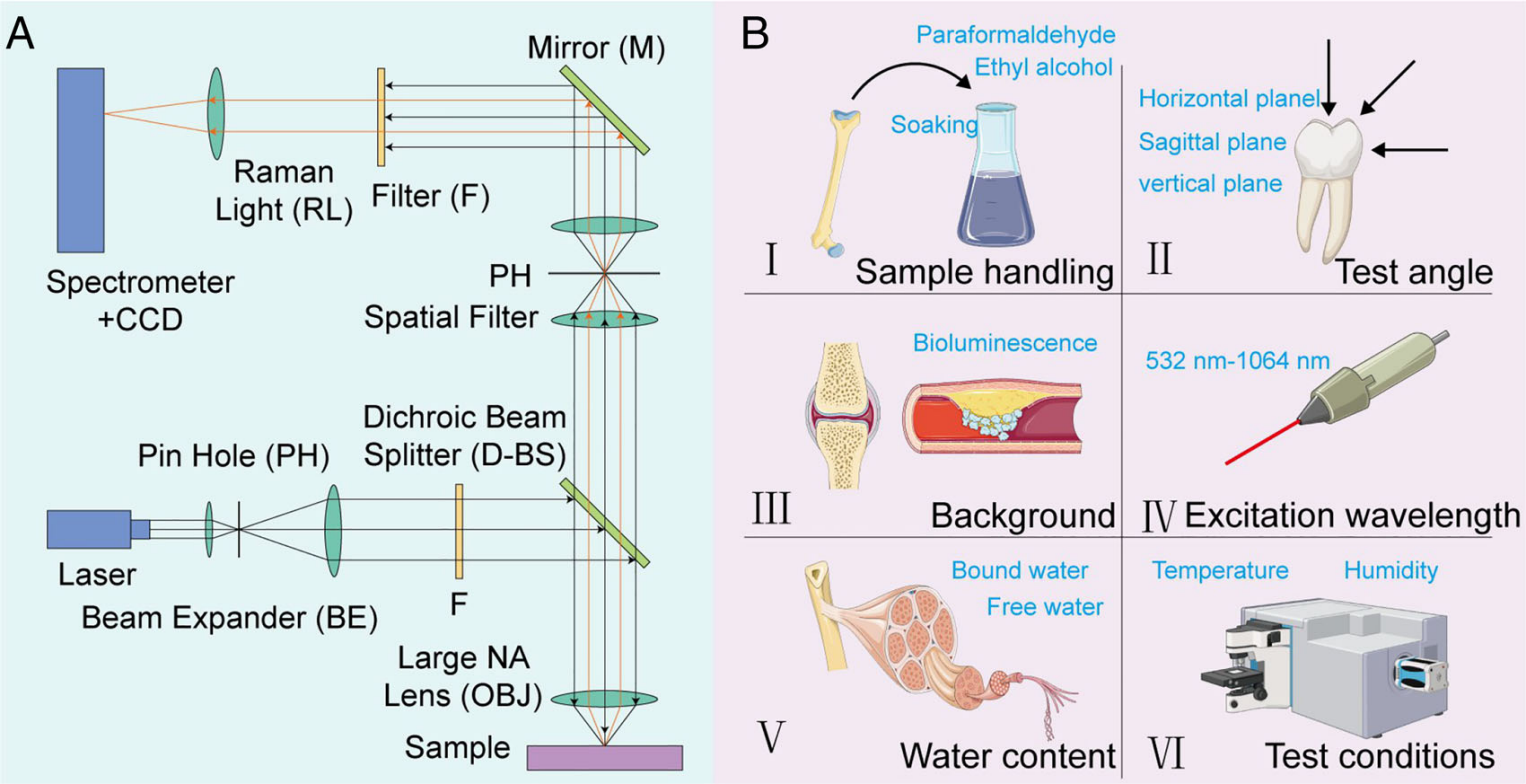
Optical construction and test conditions of the Raman spectrometer. A, Schematic diagram of the optical path of Raman spectrometer. B, The relevant study conditions of Raman spectroscopy for biomineralization.

Confocal Raman spectroscopy devices typically use an infinite correction objective to focus the pump light and a pinhole module to spatially filter the light to achieve confocal mode (Figure 2A). The pinhole transmits light from the focal plane to the detector. A diode‐pumped solid‐state laser is utilized as a monochromatic light source.^[^ ^85^, ^94^, ^95^ ^]^ A piezoelectric‐driven nanopositioner was used to position the specimen to enhance the required accuracy of the scan.^[^ ^82^, ^85^ ^]^ To detect the Raman mapping signal, a holographic imaging spectrometer with an attached CCD camera was used. In Raman spectroscopy, wavelengths of *λ* = 512, 785, 830, 980, and 1064 nm are the most common.^[^ ^96^, ^97^, ^98^, ^99^ ^]^ Excitation sources at lower visible wavelengths cause intense photoluminescence (or fluorescence) of organic macromolecules in biomineralized tissue, which can mask Raman peaks to affect the analysis of Raman spectra.^[^ ^84^, ^100^, ^101^, ^102^ ^]^ The wavelength of the excitation source is usually chosen to be *λ* = 512 or 785 nm, which will effectively avoid the occurrence of photoluminescence.^[^ ^103^, ^104^, ^105^ ^]^ The selection of excitation light power also plays an important role in the analysis of the biomineralized process (Figure 2B). When the laser power is low, the signal‐to‐noise ratio of the Raman spectrum will be poor, affecting further analysis results. When the power is high, it may damage the tissue or light quench the signal.

Important for Raman spectroscopy is the acquisition and processing of samples (Figure 2B). It can make the sample keep the original chemical composition and structure to get better results in Raman spectroscopy analysis. When bone tissue or teeth are obtained, the operation requires avoiding the use of metal instruments to avoid scratching the tissue. The acquired tissue needs to be measured for Raman spectroscopy promptly to prevent tissue denaturation, which can lead to inaccurate results.^[^ ^106^, ^107^ ^]^ If the timely measurement is not possible, the tissue can be fixed and decalcified by organic matter and placed in cryopreservation or directly in liquid nitrogen for preservation.^[^ ^108^, ^109^, ^110^, ^111^, ^112^ ^]^ It is noteworthy that during the decalcification process, the hydrochloric acid/ethylenediamine tetra‐acetic acid (HCl/EDTA) mixture removed most of the minerals. Still, it affected the secondary structure of the collagen.^[^ ^113^ ^]^ In contrast, the bones were treated with HCl alone, leaving the collagen structure well preserved in the shortest time. And the bone decalcified by CH_2_O_2_ had the highest collagen quality parameters. The interference of ethanol used to fix the tissue appears to be relatively less pronounced than that of glycerol.^[^ ^114^, ^115^ ^]^ The location of dissection, fixation, and embedding has to be considered, which can also impact the results of Raman spectroscopy analysis. During subsequent cryopreservation of the samples, the Raman spectra of the frozen bone tissue showed a significant decrease in the amide I and amide III bands, the proline residues were consistent with fresh tissue, and the mineral crystallinity decreased significantly after only one freezing.^[^ ^116^, ^117^ ^]^ The mineral carbonate content did not deviate significantly during freeze–thaw.

The direction of incident light and the direction of polarization will also have an impact on the final result. The intensity of the Raman band is not related to the *a*‐axis or *b*‐axis direction of the single hydroxyapatite crystal but only to the *c*‐axis direction.^[^ ^118^ ^]^ Both minerals and collagen fibers present in biomineralized tissues are directional.^[^ ^119^, ^120^ ^]^ Significant bands such as ν_1_ PO_4_^3−^ and amide I, which are used to determine the mineral and organic composition, are very sensitive to the direction of incident light and the direction of polarization,^[^ ^121^ ^]^ whereas bands like amide III, ν_2_ PO_4_^3−^ and ν_4_ PO_4_^3−^ are less susceptible to the influence of directional objects.^[^ ^122^ ^]^ Therefore, the incident light direction and polarization direction should be kept consistent among samples in Raman spectroscopy testing. To ensure the confidence of the results, the analysis of relevant Raman spectroscopy should be accurate and avoid errors.

### 2.3 Physicochemical study of Raman spectroscopy on biomineralization

It is known that each peak illustrated by Raman spectroscopy corresponds to a specific molecular bond vibration, which includes both the single chemical bond and the vibration of a group composed of several chemical bonds involved in biomineralization. The Raman spectral peaks in biomineralized tissues include those attributed to inorganic and organic substances.^[^ ^123^, ^124^, ^125^ ^]^ The main mineral component in teeth and bones is hydroxyapatite, which exhibits ν_1_ PO_4_^3−^, ν_2_ PO_4_^3−^, ν_3_ PO_4_^3−^, and ν_4_ PO_4_^3−^ fundamental frequency modes in the Raman spectrum (Table 1). The sharpest and most intense band, ν_1_ PO_4_^3−^, is associated with the symmetric stretching of the oxygen atom tetrahedra around the phosphorus atom, with the main peak located at 960 cm^−1^, representing the characteristic peak of hydroxyapatite.^[^ ^59^, ^126^ ^]^ Collagen plays a role in the induction of hydroxyapatite nucleation and growth in bone and tooth mineralization.^[^ ^127^, ^128^, ^129^ ^]^ The three main amide bands of collagen are amide I (1675 cm^−1^), amide II (1565 cm^−1^), and amide III (1272 cm^−1^). Among them, ν_1_ PO_4_^3−^ and amide III are susceptible to the direction of incident light and the direction of polarization. This phenomenon can be used to analyze the orientation of the crystal and collagen fibers.^[^ ^3^, ^33^, ^130^, ^131^ ^]^ In addition to the characteristic peaks of collagen and hydroxyapatite, there are other peaks, including ν_1_ CO_3_^2−^, ν_2_ CO_3_^2−^, ν_4_ CO_3_^2−^, ν(C─C), δ(CH_2_) and ν(O─H), etc. (Table 1).

**TABLE 1:** Raman spectroscopic band assignments for biomineralization.

Raman shift (cm^−1^)	Assignment	Comments	Refs.
430	ν_2_ PO_4_^3−^	Strong band (P─O stretching)	^[^ ^132^ ^]^
582	ν_4_ PO_4_^3−^	P─O bending	^[^ ^133^ ^]^
756	ν_4_ CO_3_^2−^	B‐type carbonate is very weak	^[^ ^133^ ^]^
856	ν(C─C)	Collagen proline may include δ(C─C─H)	^[^ ^120^ ^]^
876	ν(C─C)	Collagen hydroxyproline	^[^ ^134^ ^]^
880	ν_2_ CO_3_^2−^	C─O stretching	^[^ ^133^ ^]^
950	ν_1_ PO_4_^3−^	Amorphous calcium phosphate (ACP), bone mineral (P─O) phase	^[^ ^135^ ^]^
955	ν_1_ PO_4_^3−^	Octocalcium phosphate (OCP), HPO_4_^2−^	^[^ ^136^ ^]^
960	ν_1_ PO_4_^3−^	Р─О symmetric stretch	^[^ ^136^ ^]^
1030	ν_3_ PO_4_^3−^	(Overlaps with proline ν (C─C) component)	^[^ ^112^ ^]^
1070	ν_1_ CO_3_^2−^	B‐type substitution (С─О in‐plane stretch)	^[^ ^133^ ^]^
1103	ν_1_ CO_3_^2−^	А‐type substitution (С─О in‐plane stretch)	^[^ ^137^ ^]^
1181	ν(C─O─C)	Phenylalanine, tyrosine	^[^ ^90^ ^]^
1209	ω(CH_2_)	Hydroxyproline, tyrosine	^[^ ^85^ ^]^
1272	Amide III	Protein α‐helix, β‐sheet and random coils (C─N─Н stretch)	^[^ ^138^ ^]^
1375	δ(CH_2_)	Proteoglycan, CH_2_ wagging	^[^ ^134^ ^]^
1410	β(CH_2_)	Lipids, CH deformation	^[^ ^91^ ^]^
1455	δ(CH_2_)	Protein, СН_2_ wag	^[^ ^91^ ^]^
1565	Amide II	C─N─Н stretch, bending N─Н	^[^ ^116^ ^]^
1675	Amide I	C═O stretch	^[^ ^139^ ^]^
3550	ν(O─H)	O─H stretching vibration	^[^ ^90^ ^]^

Pathological mineralization, such as tumor micro mineralization, urinary stones, and atherosclerosis, is distinct from the mineral and organic composition of physiological mineralization.^[^ ^7^, ^8^, ^140^, ^141^, ^142^ ^]^ In atherosclerosis, the characteristic marker bands of the protein are located at 1181, 1209, 1272, 1410, 1565, and 1675 cm^−1^, corresponding to ν(C─O─C), ω(CH_2_), amide III, β(CH_2_), amide II and amide I, respectively (Table 1). One of the minerals belongs to carbonate apatite, which also has the characteristic peak of ν_1_ PO_4_^3−^ at 960 cm^−1^.^[^ ^143^ ^]^ Microcalcification of tumor tissue is usually due to oversized tumor tissue, resulting in deep tumors not receiving nutrients These tumor cells eventually calcify into minerals after necrosis.^[^ ^144^, ^145^ ^]^ The calcified composition is mainly that of apatite. However, due to the heterogeneity of the tumor, differences in organic matter composition can exist. The cause of urinary stones is the nucleation of insoluble microcrystals in the urethra and kidney tubules.^[^ ^64^, ^146^ ^]^ These insoluble microcrystals are various types of calcium oxalate crystals.^[^ ^147^, ^148^ ^]^ In the urinary environment, the nucleation and dissolution processes of calcium oxalate crystals are in a dynamic equilibrium, while the true stone formation process is still unknown.

### 2.4 Morphology and other characterization of biomineralization

As described above, the specific mineralization information could be readily obtained by Raman spectroscopy. Meanwhile, other characterization strategies have been also utilized to analyze the morphology and crystal morphology of the biomineralization. Scanning electron microscopy (SEM) provides nanoscale resolution of sample morphological features and, together with an energy dispersive spectrometer (EDS), it can provide quantitative elemental analysis of mineralized tissues.^[^ ^149^, ^150^ ^]^ X‐ray diffraction (XRD) is the main method for studying the physical phase and crystal structure of minerals.^[^ ^9^, ^151^, ^152^ ^]^ The crystallographic characteristics such as composition, crystalline shape, intra‐molecular bonding patterns, molecular conformation, and conformation are obtained by the diffraction phenomenon of minerals irradiated by X‐rays to different degrees. X‐ray computed tomography uses precisely collimated X‐ray beams to obtain macroscopic structural images of biomineralized tissues.^[^ ^153^, ^154^ ^]^ Multiple analysis techniques are needed to obtain comprehensive nano‐structural information on the general nature of relevant biomineralization.

## 3 BIOMINERALIZATION MECHANISM

### 3.1 Collagen

**FIGURE 3:**
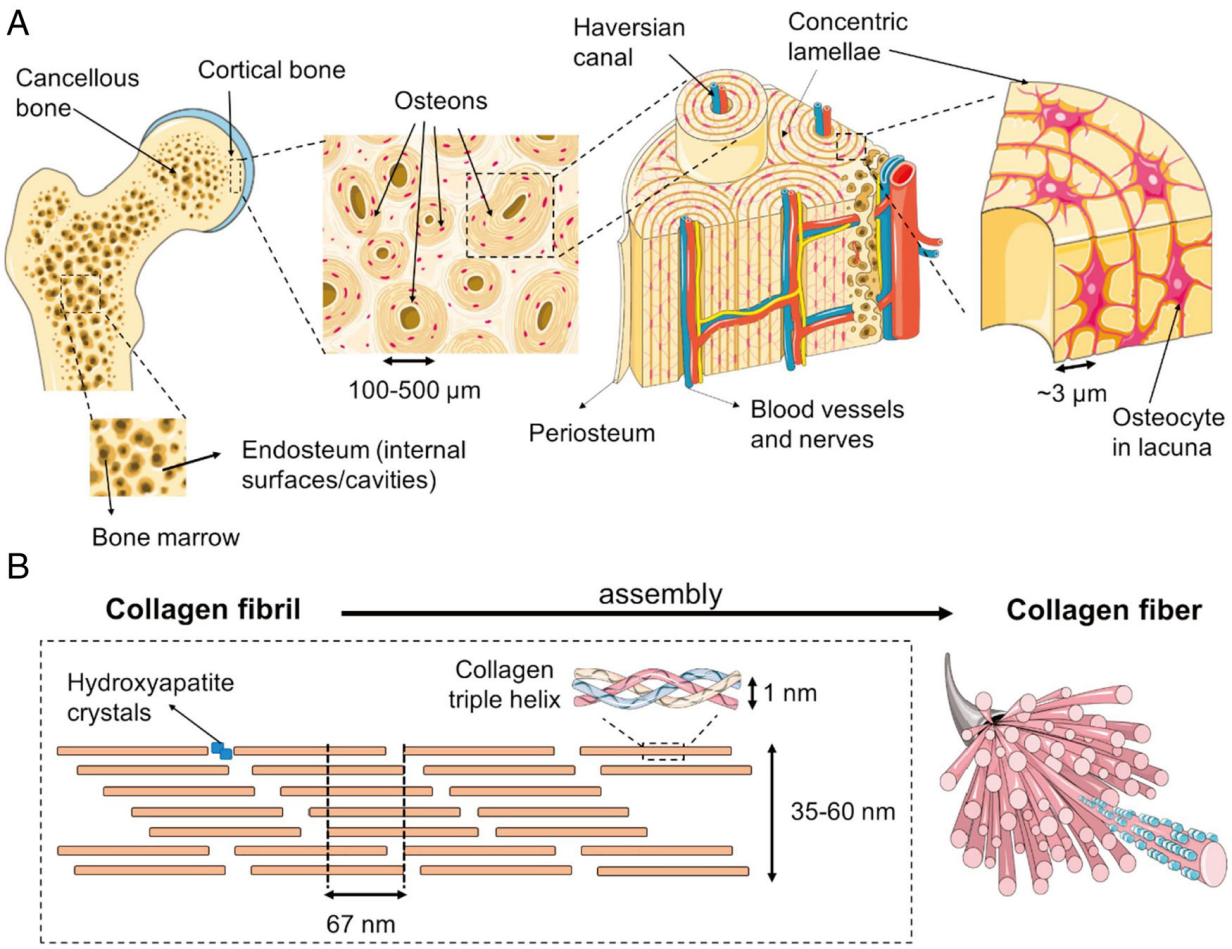
Physiological processes of bone biomineralization. A, A macroscopic‐to‐microscopic view of cancellous and cortical bone. B, Schematic diagram of the triple helix structure of self‐assembled collagen fibers in bone. Reproduced with permission.^[^ ^19^ ^]^ Copyright 2018, Elsevier.

Collagen is present in most biomineralized tissues in vertebrates, and collagen provides nucleation and crystallization sites for minerals (Figure 3A). Collagen fibers are divided into three types, types I, II, and III, which consist of glycine‐X‐Y repetitive sequences in collagen fibers (Figure 3B) where X is proline or hydroxyproline and Y is lysine or hydroxylysine. Both of which can be detected in Raman spectra. Amide I (1675 cm^−1^) and Amide III (1272 cm^−1^) bands did not change with increasing glycosylation, indicating the conserved triple helix structure of collagen fibers.^[^ ^155^ ^]^ However, significant changes in the proline may affect bone toughness. In addition, the tissues created by longitudinal fiber stacking have more tensile properties than those created by transverse fiber stacking.^[^ ^156^ ^]^ The involvement of longer collagen fibers also enhances the toughness of the bone.^[^ ^157^ ^]^ This means that when the ratio of ν_1_ PO_4_^3−^/Amide III (960/1272 cm^−1^) increases, the bones become more fragile (Table 2).

**TABLE 2:** Methodology for the study of the physicochemical properties of biomineralization by Raman spectroscopy.

Raman shift (cm^−1^)	Assignment	Method	Comments	Refs.
582/1272	ν_4_ PO_4_^3−^/Amide III	Integral	Mineral‐to‐matrix ratio (Less susceptible to tissue orientation effects)	^[^ ^158^ ^]^
960/1272	ν_1_ PO_4_^3−^/Amide III	Integral	Mineral‐to‐matrix ratio (Strongly sensitive to laser polarization)	^[^ ^105^ ^]^
1070/960	ν_1_ CO_3_^2−^/ν_1_ PO_4_^3−^	Integral	Mineral crystallinity (Carbonate‐to‐phosphate ratio)	^[^ ^159^ ^]^
1070/1675	ν_1_ CO_3_^2−^/Amide I	Integral	Represents the labile type‐B carbonate content present in the tissue matrix. (Carbonate‐to‐matrix ratio)	^[^ ^158^ ^]^
1375/1272	δ(CH_2_)/Amide III	Integral	The relative amount of proteoglycans	^[^ ^160^ ^]^
1410/1272	β(CH_2_)/Amide III	Integral	The relative amount of lipids	^[^ ^158^ ^]^
950/960	ACP/ν_1_ PO_4_^3−^	Integral	The relative content of immature hydroxyapatite	^[^ ^159^ ^]^
955/960	OCP/ν_1_ PO_4_^3−^	Integral	The relative content of immature hydroxyapatite	^[^ ^136^ ^]^
1070/1103	ν_1_ CO_3_^2−^/ν_1_ CO_3_^2−^	Integral	The relative content of A, B‐type substitution	^[^ ^136^ ^]^
1209/1181	ω(CH_2_)/ν(C─O─C)	Integral	Organic matrix properties (Hyp/Pro)	^[^ ^160^ ^]^
1070	ν_1_ CO_3_^2−^	Peak fitting	Inversely correlated with the degree of mineral crystallinity and crystallite length (Full width at half maximum (FWHM))	^[^ ^158^ ^]^
960	ν_1_ PO_4_^3−^	Peak fitting	Inversely correlated with the degree of mineral crystallinity and crystallite length (Full width at half maximum (FWHM))	^[^ ^158^ ^]^

Amide I is a characteristic peak of the collagen secondary structure, not the collagen crosslink content.^[^ ^157^ ^]^ The secondary structure formed by less collagen cross‐linking can directly show the Raman characteristic peak. It is often used as an internal standard for the relative quantification of other substances.^[^ ^161^ ^]^ Due to the different content of collagen in different biomineralized tissues, Raman mapping of Amide I and Amide III was also used to distinguish between the two, such as cartilage and cortical bone, and so on.^[^ ^162^, ^163^, ^164^ ^]^

### 3.2 Hydroxyapatite

**FIGURE 4:**
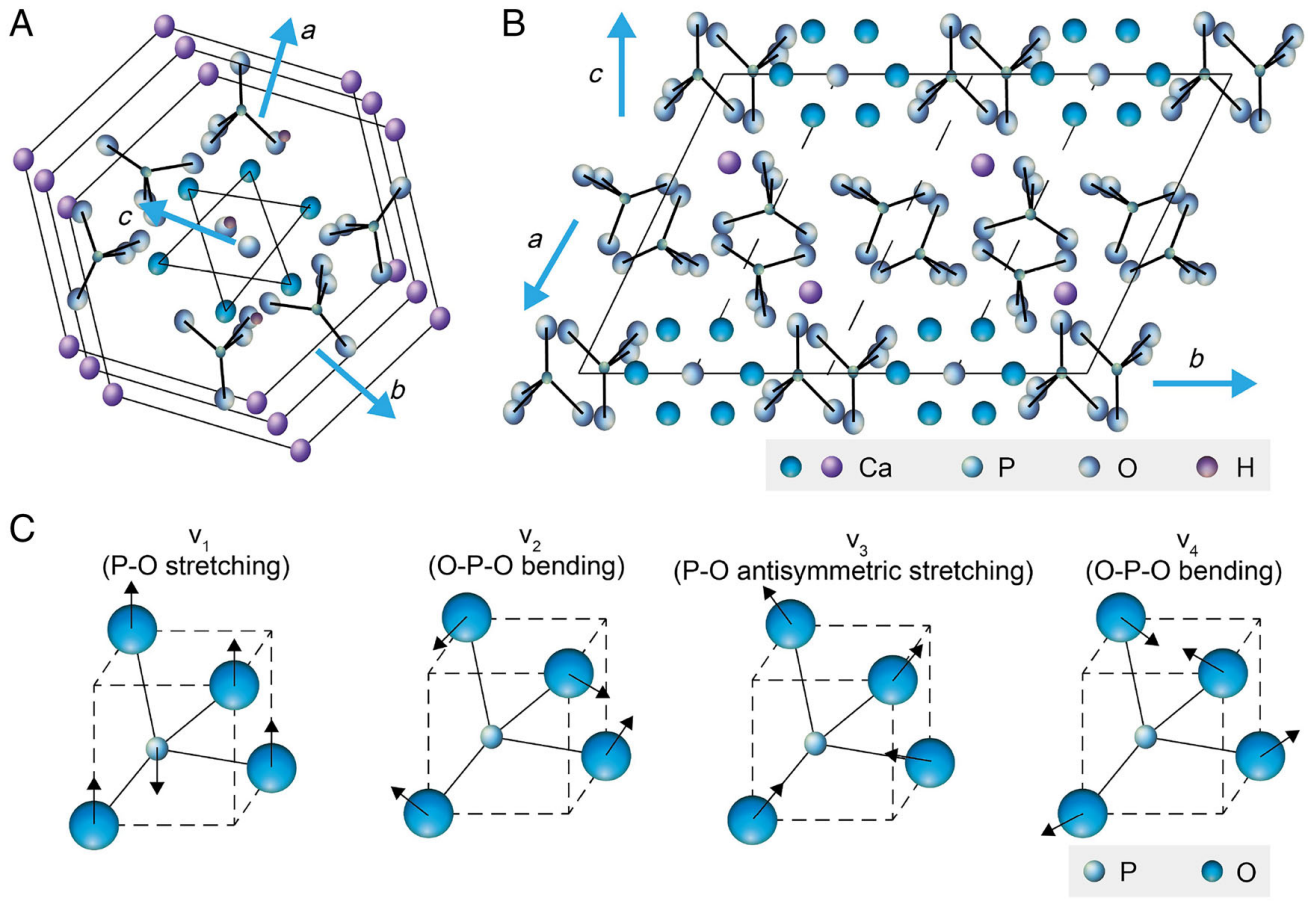
The crystal structure of hydroxyapatite. A,B, The lattice cell structure of hydroxyapatite. C) Types of hydroxyapatite vibrational modes.

Hydroxyapatite is an important component of bones and teeth in the human body. The *c*‐axis is the direction of hydroxyapatite crystal growth with an aspect ratio of up to 1000 (Figure 4A). The main mineral component in teeth and bones is hydroxyapatite, which exhibits ν_1_ PO_4_^3−^, ν_2_ PO_4_^3−^, ν_3_ PO_4_^3−^, and ν_4_ PO_4_^3−^ fundamental frequency modes in the Raman spectrum (Figure 4B,C). The orientation of this hydroxyapatite affects the characteristic peaks of ν_1_ PO_4_^3−^ in the Raman spectra of the enamel.^[^ ^122^, ^161^ ^]^ Hydroxyapatite is not directly produced during the maturation of enamel or bone.^[^ ^136^, ^165^, ^166^ ^]^ Rather, nucleation and crystallization of amorphous calcium phosphate (ACP) on collagen fibers. Over time, the crystalline phase transforms into calcium phosphate (OCP), eventually forming mature hydroxyapatite crystals.^[^ ^32^, ^136^, ^159^, ^167^, ^168^ ^]^ During this process, ν_1_ PO_4_^3−^ begins to red‐shift from 950 to 960 cm^−1^ (Table 2).

During the maturation of the enamel, ions (Mg^2+^, Cl^−^, F^−^, OH^−^, CO_3_^2−^) are constantly embedded in the lattice of hydroxyapatite crystals.^[^ ^132^, ^169^, ^170^, ^171^ ^]^ Ionic doping is generally achieved by replacing hydroxyl groups with hydroxyapatite. The intensity or frequency of phosphate and carbonate vibrational peaks can be used to quantify the type and extent of halogen ion substitution.^[^ ^172^, ^173^ ^]^ Then, ν_4_ PO_4_^3−^ in hydroxyapatite crystals, unlike ν_1_ PO_4_^3−^, is not affected by crystal orientation, and its half‐peak width is often used to analyze crystal crystallinity or is used as an internal reference.^[^ ^174^, ^175^, ^176^ ^]^ The half‐peak width of ν_1_ PO_4_^3−^, the ratio of ν_1_ CO_3_^2−^/ν_4_ PO_4_^3−^ or the ratio of ν_1_ CO_3_^2−^/ν_1_ PO_4_^3−^ have also been studied as indicators of the degree of hydroxyapatite crystallization.^[^ ^162^, ^174^, ^175^, ^176^ ^]^ Interestingly, the higher crystallinity of hydroxyapatite crystals leads to an increase in hardness and a decrease in toughness.

### 3.3 Carbonate

The biomineralization process of calcium carbonate is similar to that of hydroxyapatite, both occurring by interaction with biological substrates. Mostly as amorphous calcium carbonate (ACC), aragonite, and calcite occur in the shells of crustaceans and the teeth of sea urchins.^[^ ^60^, ^61^, ^177^ ^]^ A portion of carbonate is also present in vertebrate enamel and bone. During the maturation of hydroxyapatite, carbonate ions replace the hydroxyl or phosphate in it, thus changing the stability and solubility of the mineral phase. The embedding of carbonate in the lattice causes a significant increase in crystal solubility and deterioration of crystallinity.^[^ ^178^, ^179^, ^180^ ^]^ The result shown in the Raman spectrum is an increase in the ratio of ν_1_ CO_3_^2−^/ν_1_ PO_4_^3−^, indicating a decrease in crystallinity (Table 2). A‐type carbonate (OH substituted by CO_3_^2−^) and B‐type carbonate (PO_4_^3−^ substituted by CO_3_^2−^) are both present in enamel and bone, but the exact spatial distribution is unknown.^[^ ^133^, ^181^ ^]^ It is worth affirming that the content of B‐type carbonate dominates and is associated with increased B‐type carbonate substitution (the ratio of B‐type/A‐type increased.) and deterioration in the mechanical properties of biomineralized tissues.^[^ ^182^, ^183^, ^184^ ^]^ The increase in B‐type substitution also leads to an increase in the half‐peak width of ν_1_ PO_4_^3−^, indicating an increase in hydroxyapatite crystal defects.

### 3.4 Lipids

Lipids play a certain role in mineralization, and mineralization‐inducing molecules are very important in atherosclerosis.^[^ ^53^, ^158^, ^185^ ^]^ Atherosclerosis is an accumulation of intimal plaques that appear in the intima of the arteries, and the plaques consist mainly of lipoproteins, oxidized lipoproteins, various cellular debris, and finally, groups of apatite deposits.^[^ ^143^, ^186^ ^]^ The apatite formed by calcification of the medial arteries of the arterial intima is embedded in the lipid deposits.^[^ ^126^ ^]^ There are individual and regional differences in atherosclerosis, which makes it impossible to understand the pathogenesis.

### 3.5 Other proteins

**FIGURE 5:**
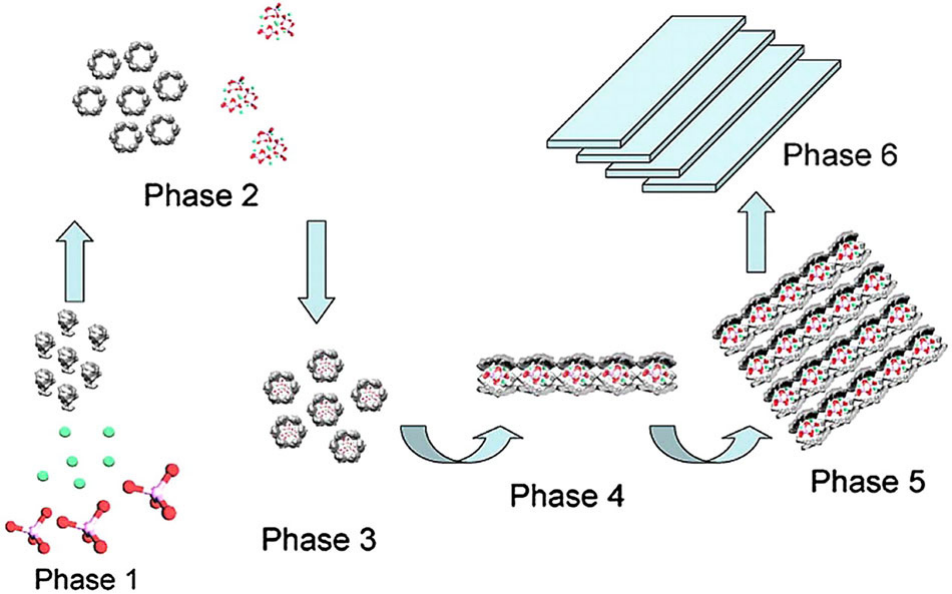
Amelogenin‐based mineral nucleation processes. The amelogenin assemblies manipulate the arrangement of prenucleation clusters into organized mesostructures. Reproduced with permission.^[^ ^195^ ^]^ Copyright 2020, National Academy of Science.

A portion of the vertebrate body is biomineralized by proteins other than collagen fibers. The organic matter in the enamel consists of AMBN, AMELX, ENAM, MMP20, ALP, and KLK4. When AMELX is secreted by ameloblasts to the outside of cells, it exists in the form of a monomer or nano aggregate.^[^ ^43^, ^44^, ^187^, ^188^ ^]^ The inside of nano aggregate is a hydrophobic core, and the outside is a hydrophilic retractable fragment.^[^ ^189^, ^190^, ^191^, ^192^ ^]^ In dependence on temperature, pH, and protein concentration, AMELX self‐assembles to form a linear morphology that provides nucleation sites for hydroxyapatite (Figure 5).^[^ ^193^, ^194^, ^195^ ^]^ This linear morphology may be the key to controlling the directional growth of hydroxyapatite.^[^ ^196^, ^197^, ^198^ ^]^ AMBN and ENAM are all present in enamel nucleation and crystallization, but their specific roles are not yet clear.

Interestingly, AMBN, AMELX, and ENAM were not found in the mature enamel. It was found that the expression and secretion of MMP20 and KLK4 are spatiotemporal throughout the process of enamel mineralization.^[^ ^28^, ^38^, ^199^ ^]^ MMP20 and KLK4 induce the degradation of enamel matrix proteins, providing space for hydroxyapatite crystal growth and making enamel harder.^[^ ^40^, ^199^ ^]^ When Raman spectroscopy is used to analyze enamel, the Amide I, II, and III bond is more of a characteristic peak of enamel matrix protein.^[^ ^58^, ^200^, ^201^, ^202^ ^]^ A typical example is the markedly enhanced intensity of the Amide III in enamel after MMP20 knockdown, indicating that the absence of MMP20 expression results in enamel matrix proteins being blocked in the enamel.^[^ ^154^ ^]^

### 3.6 Other minerals

Calcium oxalate stones are formed under pathological conditions. Its chemical formula is CaC_2_O_4_ or Ca (COO)_2_, fundamentally different from hydroxyapatite and carbonate.^[^ ^146^, ^203^, ^204^, ^205^ ^]^ The Raman characteristic peaks of calcium oxalate are also distinct. Characteristic bands for calcium oxalate monohydrate are assigned to the C─O asymmetric stretch (1630 cm^−1^), C─O symmetric stretch (1485 and 1460 cm^−1^), and C─C stretch (895 cm^−1^), and O─C─O in‐plane bending (500 cm^−1^).^[^ ^148^, ^206^, ^207^, ^208^ ^]^ The pathological environment is more complex, and the embedding of different organic matter also causes changes in the Raman spectroscopy of biomineralization. The exact trend of the changes is not clear. In addition to calcium oxalate stones, there are xanthine stones, cystine stones, ammonium magnesium phosphate stones, uric acid (urate) stones, and calcium phosphate stones.^[^ ^209^, ^210^, ^211^ ^]^

## 4 PHYSIOLOGICAL PROCESSES AND DISEASE DIAGNOSIS

### 4.1 Enamel mineralization

**FIGURE 6:**
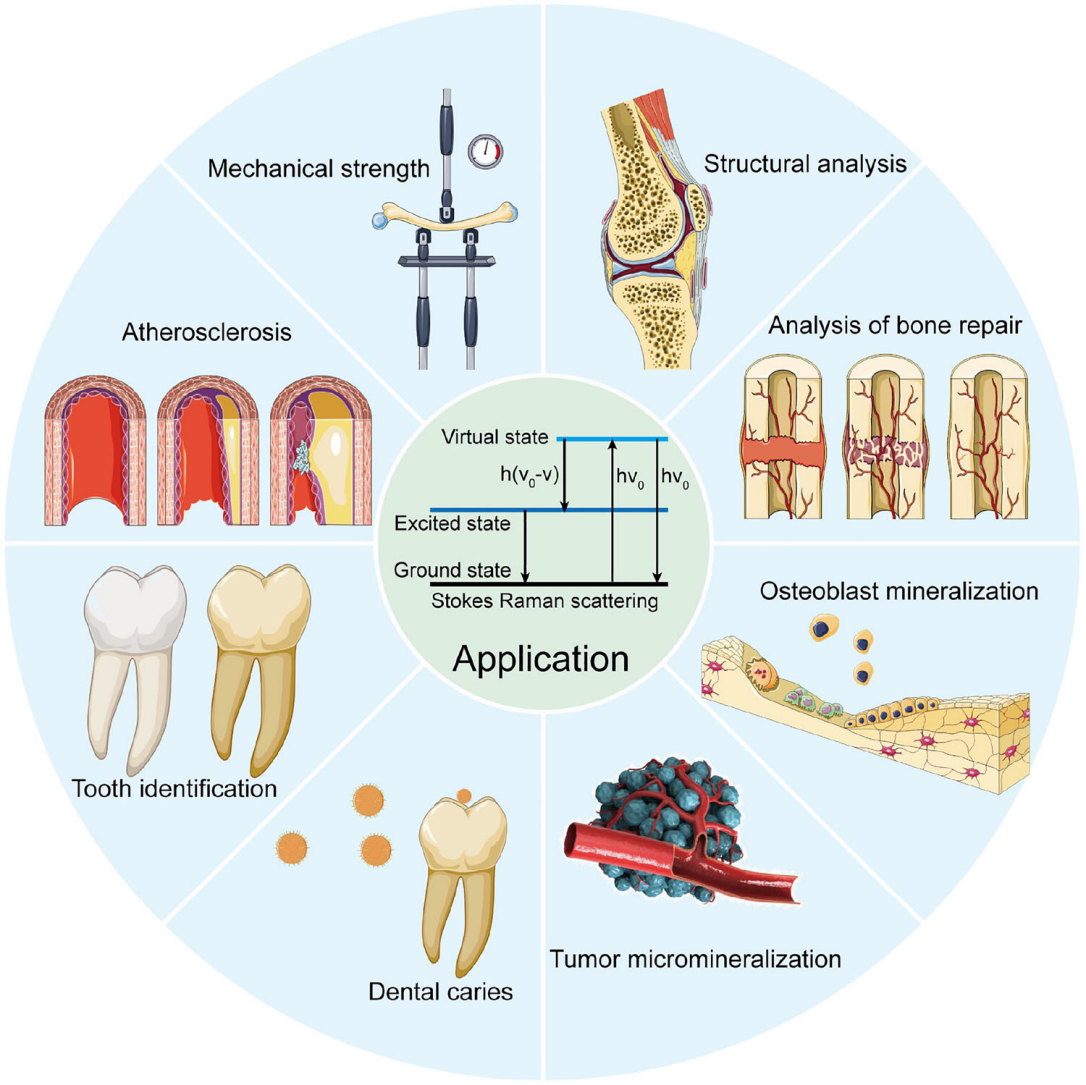
The application of molecular vibrational spectroscopy in biomineralization studies.

Teeth are biomineralized body tissues exposed to the external environment, which differs from bones. Enamel is more susceptible to exogenous damage from bacteria, food debris, etc., causing plaque, dental caries, periodontitis, and other diseases in the teeth.^[^ ^200^, ^212^ ^]^ Meanwhile, the enamel is produced under the regulation of ameloblasts, which undergo programmed apoptosis after enamel maturation. If there is faulty signaling by ameloblasts in the regulation of enamel maturation, it can lead to irreversible amelogenesis imperfect.^[^ ^2^, ^164^ ^]^ Therefore, studying exogenous and endogenous factors that affect the physicochemical properties of enamel mineralization is an essential basis for maintaining enamel function (Figure 6).

The full‐scale Raman imaging (≈7 mm in diameter) covering the cross‐sectional area of the laser beam can be realized to illustrate biomineralization by using a high‐power laser.^[^ ^213^ ^]^ The laser can be guided to the sample without passing through the objective lens, which greatly saves the time of Raman scanning. The full‐scale Raman image based on mineral intensity distribution can be used to recognize and distinguish enamel and dentin, as well as the normally healthy and carious enamel.^[^ ^213^ ^]^ Polarization resolution hyperspectral stimulated Raman imaging technology can analyze the direction change of biomolecules in dental caries on the submicron scale. The depolarization rate of the microscope objective used is ≈0.005, which is much smaller than the difference between the depolarization rates of normal enamel and caries, allowing the marking of caries.^[^ ^56^ ^]^ Polarized Raman spectroscopy revealed that healthy enamel exhibited strong Raman polarization anisotropy, while all early caries showed low Raman polarization anisotropy.^[^ ^214^ ^]^ The Raman spectroscopy generated by ν_1_ PO_4_^3−^ vibrations was strongly polarized, while the polarization dependence was weaker in the caries region. This difference in the degree of polarization anisotropy of Raman spectra becomes a distinct marker to distinguish early caries from sound enamel. Meanwhile, Raman spectroscopy has also been used in combination with other techniques for the synergistic diagnosis of dental caries, such as optical coherence tomography.^[^ ^57^ ^]^

**FIGURE 7:**
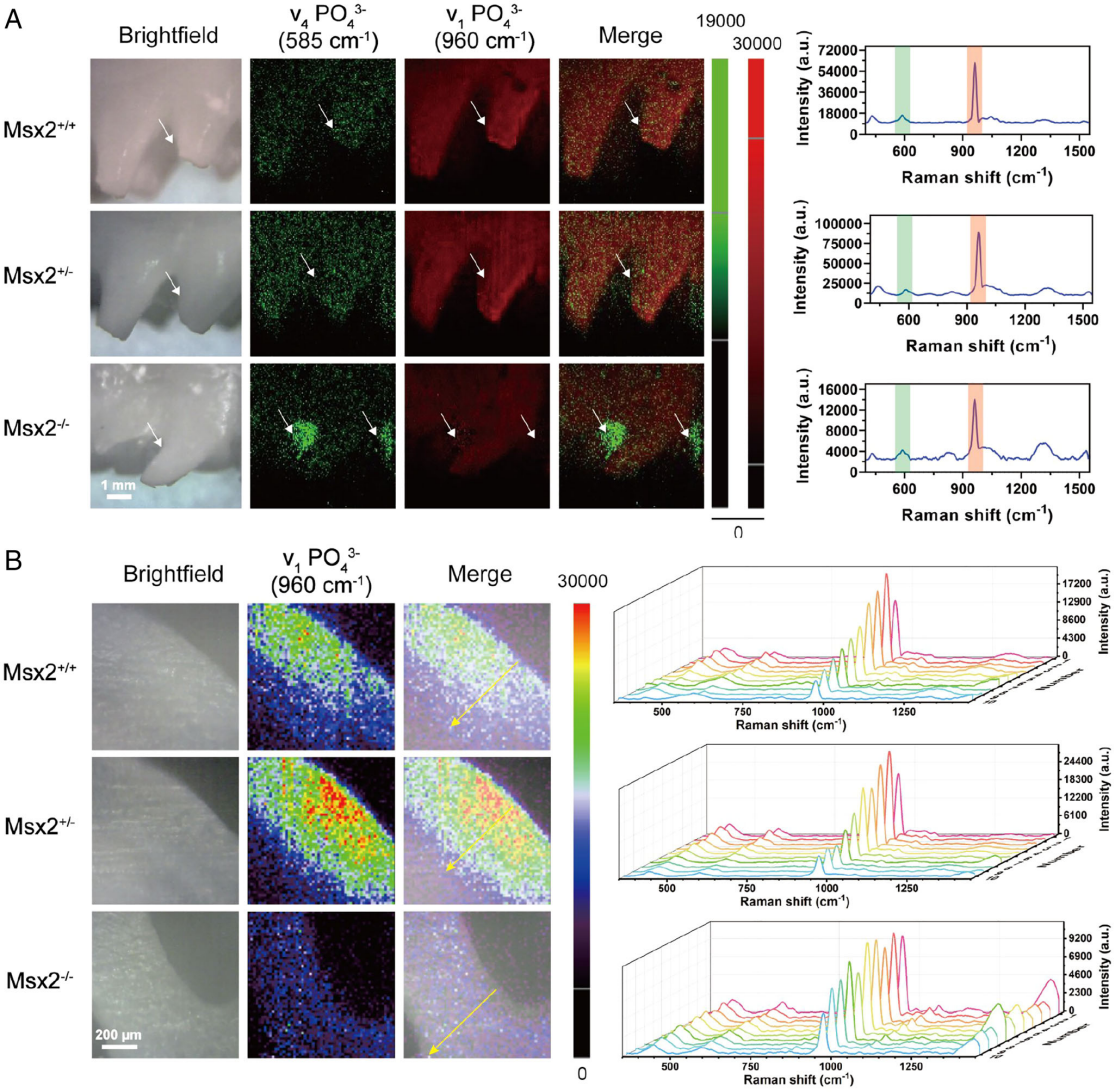
Raman spectra of tooth enamel and their correlation mapping. A, Raman spectroscopic imaging of enamel surfaces. B, Raman analysis of enamel sagittal plane of molars. Reproduced with permission.^[^ ^105^ ^]^ Copyright 2021, Wiley‐VCH.

Pathological enamel damage causes a change in the crystal structure of hydroxyapatite, and the result is a change in the characteristic peaks of Raman spectroscopy.^[^ ^58^, ^201^, ^215^, ^216^ ^]^ Raman spectroscopy was especially used to analyze the relationship between Msx2 gene expression in ameloblasts and enamel.^[^ ^105^ ^]^ There were differences in enamel composition after knocking out Msx2 in ameloblasts. Figure 7A shows the Raman images reflecting the phosphate vibration modes ν_1_ PO_4_^3−^ and ν_4_ PO_4_^3−^ in enamel. The position of ν_4_ PO_4_^3−^ in enamel after Msx2 knockout is uneven, which may be due to the disorder of spatial distribution during mineralization.^[^ ^105^ ^]^ And it will affect the content of hydroxyapatite in enamel (Figure 7B). After periodontitis treatment, the main structural changes in tooth tissue occur in the cementum. The main reasons for the structural changes of cementum are microbial infection, susceptibility, and periodontal pocket susceptibility. The alteration of the characteristic peaks of Raman spectra caused by this phenomenon was used by the Timchenko group to identify the effect of the treatment of periodontitis.^[^ ^200^ ^]^ Marco Antonio diagnosed dental fluorosis by improving the classification algorithm.^[^ ^217^ ^]^ It was found that the b‐type carbonate content increased with fluorosis severity. And the specificity of the PCA‐LDA model, that is, the combination of principal component analysis with linear discriminant analysis, for different fluorosis severity groups was higher than 93%, which could be utilized to effectively distinguish different degrees of fluorosis.^[^ ^217^ ^]^

Raman spectroscopy has unique advantages in dental analysis, such as the ability to resolve crystal structure, and content and analyze crystal orientation. Both the study on the characteristic Raman spectroscopy of biomineralization and the improvement of the Raman spectrometer to upgrade algorithms will enhance the potential of Raman spectroscopy in diagnosing dental diseases.

### 4.2 Bone mineralization

The analysis of bones by Raman spectroscopy can reveal much more detailed information on relevant biomineralization. For example, the structural information of the femoral shaft, the structural information at the joints, the spatial distribution of the skull, the trabecular structure could be readily obtained, etc.^[^ ^52^, ^174^, ^218^, ^219^ ^]^ The effects of different diseases on bone physicochemical properties were analyzed by Raman spectroscopy (Figure 6). It is observed that Raman spectroscopy can provide specific mechanical information about bones, predict the physiological age of organisms and the risk of fracture, etc., through the combination of logical operation and algorithm.

**FIGURE 8:**
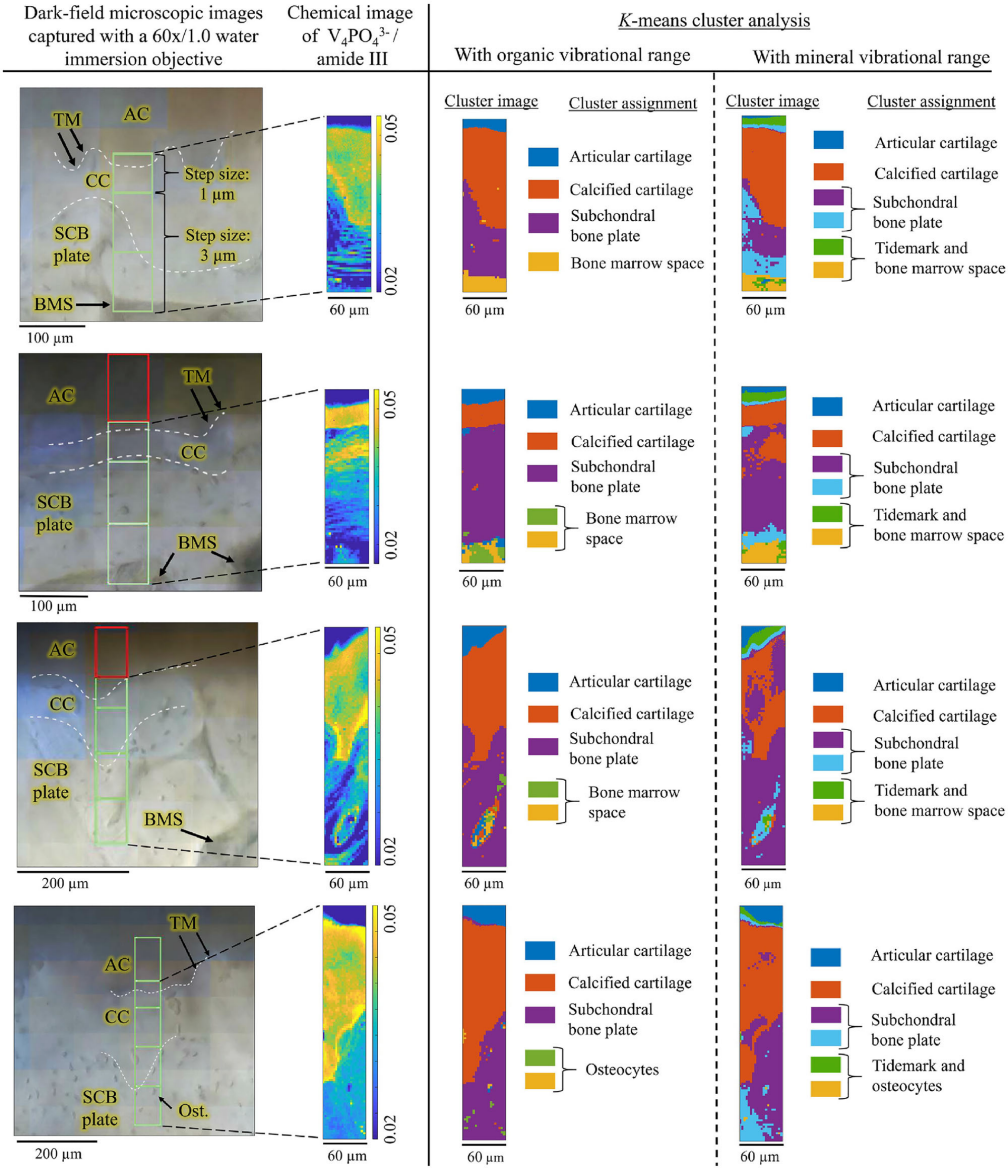
Raman spectra of bone and their correlation mapping. Comparison between the dark‐field microscopic images, the chemical maps of mineral‐to‐matrix ratio (ν_4_ PO_4_^3−^ / amide III), and the KMC analysis for four samples. Reproduced with permission.^[^ ^158^ ^]^ Copyright 2020, Elsevier.

Raman spectroscopy was also used to study the effect of the severity of osteoarthritis on the biological composition of the human tibial plateau osteochondral junction.^[^ ^158^ ^]^ Through multivariate cluster analysis, calcified cartilage, subchondral bone plate, calcified cartilage, and non‐calcified cartilage were identified (Figure 8).^[^ ^158^ ^]^ The unsmooth bones lead to the difficulty of Raman imaging. Anders et al. solve the challenge brought by the inherent topology of this unique biological system by using the real‐time focusing and tracking technology of continuous closed‐loop feedback to optimize laser focusing.^[^ ^220^ ^]^ In situ, analysis of organic and inorganic components of the biomineralization is possible despite surface height deviations of more than 100 μm in the femur. There are also some Raman spectroscopic studies focusing on bone diseases and the analysis of bone complications caused by other conditions, such as osteoporosis and type‐II diabetes.

The mineral apatite of cortical bone tissue and bone in healthy and ovariectomized (OVX)—induced osteoporosis in female mice was studied.^[^ ^221^ ^]^ It was found that the lesion did not undergo significant amorphization, but the relative content of organic matter changed. Pankaj et al. show that type‐II diabetes and related therapeutic drugs can harm bone quality through Raman spectroscopy.^[^ ^222^, ^223^ ^]^ Some molecular biologists have studied the effects of different gene expressions on bone quality in mice by Raman spectroscopy, such as low‐density lipoprotein receptor (LDLr) and plastin3 (PLS3).^[^ ^48^, ^224^ ^]^ From the level of disease to molecular biology, Raman spectroscopy is more commonly utilized to obtain the distinct structural information of the bone.

Brittleness, toughness, and mechanical strength of bone are important evaluation criteria for bone quality. Ozan et al. found that while the elastic deformation ability decreased, the increase of mineralization, crystallinity, and substitution degree of B‐type carbonate was significantly related to the decrease of elastic deformation ability with age.^[^ ^183^ ^]^ Osteogenesis imperfecta (OI) is a genetic disorder that manifests on a macroscopic scale as an increase in bone fragility. The Raman spectroscopy results showed a higher mineral‐matrix ratio and lower crystallinity in OI samples, suggesting that OI samples have smaller but more abundant mineral crystals that can lead to increased bone fragility.^[^ ^225^ ^]^ Besides, it was found that a decrease in the low‐frequency component of the amide III band and an increase in the high‐frequency component of the amide I band were found, indicating the rupture of collagen crosslinks.^[^ ^226^ ^]^ This breakage of collagen's secondary structure is affecting the mechanical properties of the bone.^[^ ^49^ ^]^ It has been validated in mouse, rabbit, and human disease models.^[^ ^49^, ^157^, ^227^, ^228^, ^229^, ^230^, ^231^ ^]^ Interestingly, based on the difference in bone quality caused by disease, some scholars took the Raman spectrum as the input of linear discriminant analysis (LDA) and evaluated that the linear support vector machine (LSVM) algorithm can successfully identify renal bone dystrophy.^[^ ^232^ ^]^

Although Raman spectroscopy can achieve bone quality assessment and mechanical performance analysis through deep learning and classification algorithms, the calculation results are unreliable due to the small sample size. Secondly, the current research on deep learning and classification algorithms for diagnosing minerals is relatively simple. To improve the accuracy of Raman spectroscopy in evaluating bone quality, more accurate deep learning and classification algorithms need to be developed.

### 4.3 Atherosclerosis

**FIGURE 9:**
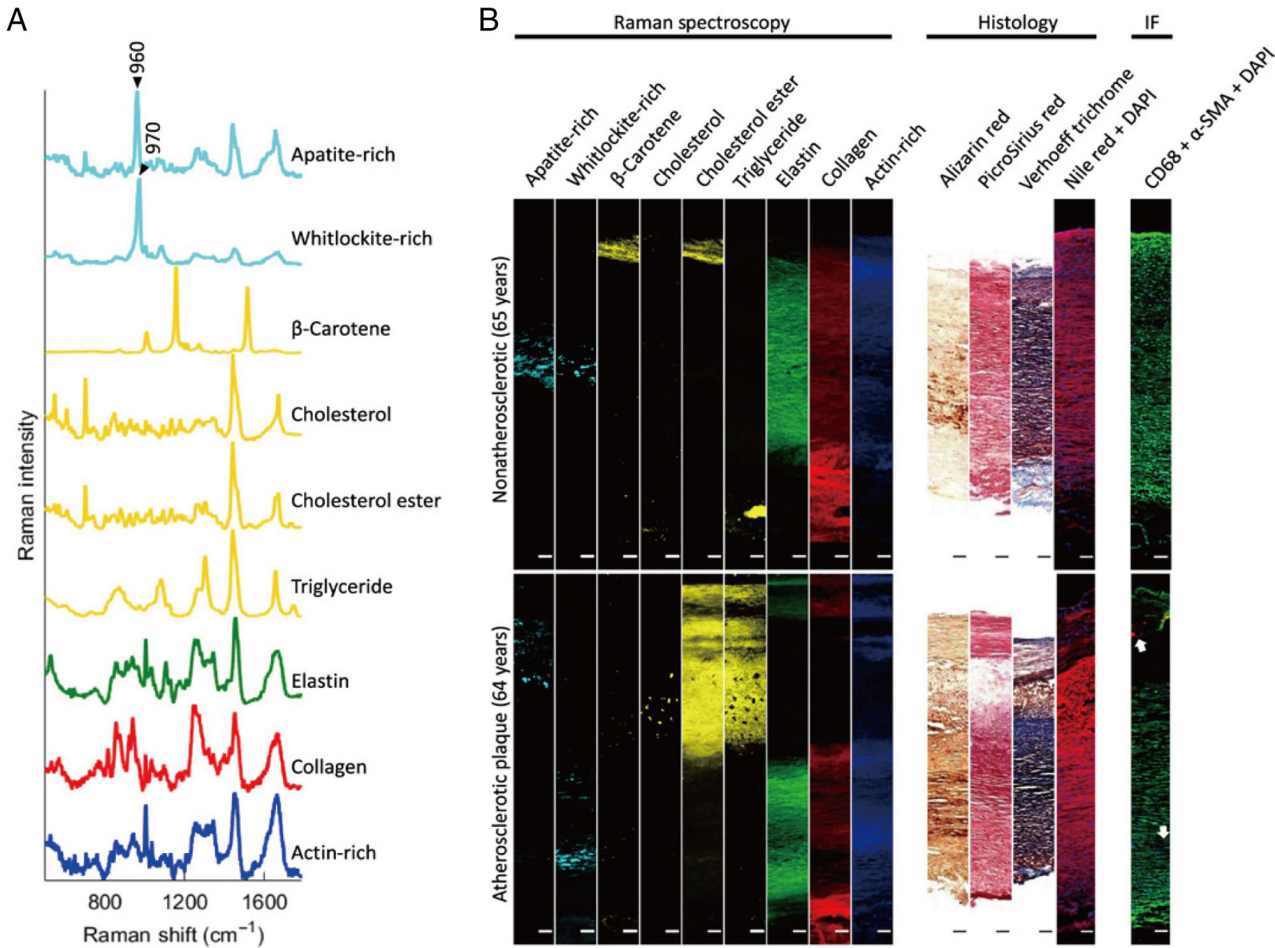
Raman spectra of atherosclerosis and their correlation mapping. A, Representative Raman spectra rich in specific aortic components. B, Representative Raman mapping images, associated histology, and immunofluorescence. Reproduced with permission.^[^ ^143^ ^]^ Copyright 2017, American Association for the Advancement of Science.

The progression of atherosclerosis depends on the amount of lipid accumulation in the intima of the arteries. The increased amount of lipid accumulation creates a risk of sudden rupture or rupture of plaques characterized by high extracellular lipid content, abundant macrophages, small amounts of smooth muscle cells, and relatively low concentrations of collagen and glycosaminoglycans.^[^ ^7^, ^233^, ^234^ ^]^ This ultimately results in the crystallization of mineral crystals. Raman spectroscopy can accurately quantify the relative amounts of calcium salts, cholesterol, triglycerides, and phospholipids in arterial tissue (Figure 9A). There are large differences in the construction of conventional atherosclerotic mouse models, while the sclerotic plaques are not easily localized. And the differences in Raman spectroscopy results in that cause atherosclerotic lesion sites suggest that the causes of atherosclerosis are diverse.^[^ ^235^, ^236^, ^237^ ^]^ Apolipoprotein E/low‐density lipoproteins (ApoE/LDLR) knockout was used as an indicator for the construction of atherosclerosis model mice.^[^ ^7^, ^62^ ^]^ The Raman spectroscopy imaging method was adjusted to distinguish the main biomolecules present in this atherosclerotic tissue to reveal the relationship between apatite, cholesterol, and triglycerides (Figure 9B).^[^ ^143^, ^238^, ^239^ ^]^ Besides, Raman spectroscopy was used to analyze the pharmacological effects of drugs in the atherosclerotic plaque in situ, providing a strong basis for the development of drugs for atherosclerosis treatment.^[^ ^235^, ^240^ ^]^

In addition to the analysis of the causes of atherosclerosis, Raman spectroscopy can be also utilized as a diagnostic tool for atherosclerosis due to the specific chemical composition of atherosclerotic plaques. Stimulated Raman scattering microscopy combined with second harmonic generation microscopy effectively distinguishes between cholesterol monocrystals, aliphatic lipids, structural proteins of the tissue matrix, and other condensed structures.^[^ ^241^, ^242^ ^]^ Some researchers reported a fiber‐optic Raman spectroscopy method to achieve in vivo atherosclerosis detection with a specificity and sensitivity of 79% and 85%.^[^ ^236^, ^243^ ^]^ To increase the detection time and accuracy of atherosclerosis, techniques such as fluorescence lifetime imaging and fluorescence imaging are used in combination with Raman spectroscopy for lesion site identification.^[^ ^239^, ^244^, ^245^, ^246^ ^]^

Atherosclerosis is highly individualized, which greatly limits the study of its triggering mechanisms. Moreover, the presence of hemoglobin leads to strong tissue autofluorescence and interference with Raman spectroscopy results. All these problems need to be addressed in subsequent studies.

### 4.4 Urinary calculi

The formation of urinary calculi is a pathological biomineralization process that may be induced by endogenous or exogenous factors. Such as gender, genetics, diet, water intake, and occupation.^[^ ^207^ ^]^ Among the types of urinary calculi are calcium oxalate dihydrate (COD), magnesium ammonium phosphate hexahydrate (MAPH), calcium oxalate monohydrate (COM), and calcium hydrogen phosphate dihydrate (CHPD), Penta‐calcium hydroxy‐triphosphate (PCHT), and uric acid (UA).^[^ ^209^ ^]^ Proteins in urinary stones cannot be detected by Raman spectroscopy due to their fluorescence background. However, carbonate and calcium oxalate, which account for less than 5% of the total stones, can be detected.^[^ ^247^ ^]^ The COD spectrum shows O─H stretching vibration at 3264^−1,^ and the C═O vibration and C─O symmetric stretching lead to a clear band change.^[^ ^8^, ^63^, ^248^ ^]^ The Raman spectra of COM showed that the presence of weaker peaks at 1631 cm^−1^ is caused by C─O asymmetric stretching, and the peak at 896 cm^−1^ is caused by C─C stretching. 502 cm^−1^, 1462 cm^−1,^ and 1473 cm^−1^ peaks are caused by O─C─O surface bending.^[^ ^8^, ^147^, ^210^, ^249^ ^]^ The Raman spectrum of MAPH is dominated by the P─O vibration peak, with an obvious peak at about 950 cm^−1^.^[^ ^250^ ^]^ The weak peak at 431 cm^−1^ is due to the phosphate band in magnesium ammonium phosphate (guano stone). The vibration peak at 584 cm^−1^ is caused by the O─P─O symmetric bending mode.

**FIGURE 10:**
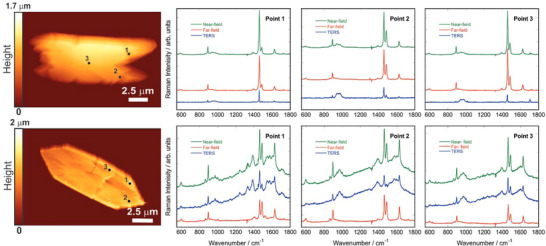
Characterization of calcium oxalate monohydrate. AFM images of COM and Raman spectra (selected spots) adsorbed p‐OPN on COM faces. Reproduced with permission.^[^ ^252^ ^]^ Copyright 2012, American Chemical Society.

The minerals of urinary stones are different, resulting in differences in the characteristic peaks of the Raman spectra, which is the main basis on which Raman spectra can be effectively analyzed and classified. Raman spectroscopy and laser‐induced breakdown spectroscopy (LIBS) were combined to evaluate the chemical composition of different classes of urinary stones. LIBS explores elemental features that complement the molecular details of the samples and improve the accuracy of the analytical results of urinary stones.^[^ ^251^ ^]^ Tip‐enhanced Raman spectroscopy (TERS) was used to enhance the characteristic peaks of proteins in urinary stones, providing information on the surface specificity of the phosphoprotein osteopontin (OPN) adsorption on the surface of COM crystals (Figure 10).^[^ ^252^ ^]^ It was revealed that competition and inhibition of COM formation by OPN plays a key role in COM formation during ectopic biomineralization of calcium oxalate kidney stones.^[^ ^252^ ^]^ Although there are significant differences in urinary calculi, which can provide effective classification information, the causes of urinary calculi need to be further studied. This is a prerequisite for providing a prognosis after the diagnosis of urinary calculi.

### 4.5 Tumor tissue microcalcifications

Tumor tissue microcalcifications are classified into two types: Type I is calcium oxalate dihydrate, and Type II is hydroxyapatite.^[^ ^253^ ^]^ Type I microcalcifications are diagnosed as benign, while Type II is considered malignant. The overall biochemical composition of the microcalcifications was used to differentiate the malignancy of the tumor tissue.^[^ ^145^ ^]^ It is noteworthy that these tumor microcalcifications are usually seen in breast cancer and that more mineral crystals increase with time.^[^ ^74^ ^]^ Tissue microcalcifications were also found in chondrogenic tumors and human skin pilomatrixoma.^[^ ^254^, ^255^ ^]^

**FIGURE 11:**
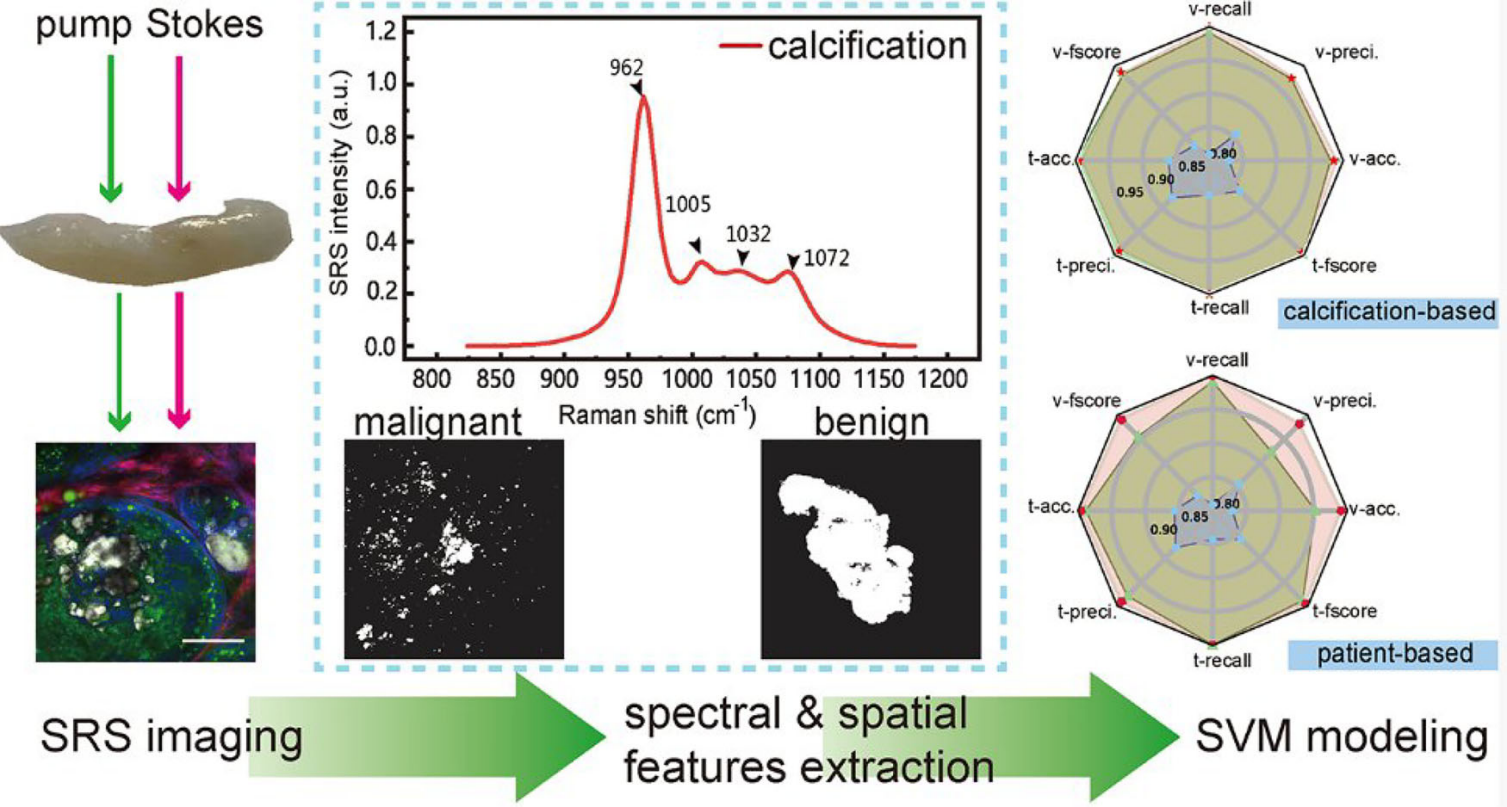
Diagnosis of microcalcifications in tumors. With the spectral and spatial domain analysis, the stimulated Raman scattering (SRS) microscopy extracts both the chemical and morphological features of the microcalcifications. Reproduced with permission.^[^ ^256^ ^]^ Copyright 2021, American Chemical Society.

Coherent anti‐stokes Raman spectroscopy was used to image breast cancer tissue based on differences in microcalcifications types.^[^ ^142^ ^]^ Hyperspectral stimulated Raman scattering spectroscopy (SRS) was used to enhance the imaging sensitivity and resolution of tumor microcalcifications, which improved the accuracy of benign and malignant tumor classification to 98.21% with the synergy of a support vector machine (SVM) based classification algorithm (Figure 11).^[^ ^144^, ^256^ ^]^ Alternatively, imaging and diagnosis of tumor microcalcifications can be achieved by introducing shell‐isolated nanoparticle‐enhanced Raman scattering (SHINERS) probes.^[^ ^257^ ^]^ In addition to the improvement and enhancement of detection methods, the study of classification algorithms, with examples like decision tree classification, k‐nearest neighbor (k‐NN), SVM analysis, PCA, and LDA, is also crucial.^[^ ^67^, ^258^ ^]^

## 5 CONCLUSION

In summary, we have reviewed the biomineralization process and relevant physicochemical properties, nano‐structure, and spatial distribution. The research progress and possible mechanism of enamel and bone mineralization, atherosclerosis, urinary calculi, and tumor microcalcification have been exploited and illustrated from the perspective of Raman spectroscopy. The physiological and pathological mineralization could be further explored for the promising diagnosis of biomineralization‐related diseases. The bio‐responsive and pathological‐microenvironmental stimulated biomineralization could readily produce in situ fluorescent and magnetic nanocrystals, which can be further utilized for multimodal tumor imaging and targeted therapy.

Raman spectroscopy has unique advantages in studying biomineralization and can facilitate the revealing of the specific physicochemical properties for relevant biomineralization processes. Further studies could be explored in the future for combining Raman spectroscopy and engineered bio‐responsive biomineralization to advance its biomedical applications for the theranostics of clinical diseases. This may provide new strategies for the early diagnosis and treatment of biomineralization‐related diseases like some heart diseases and cancers.

## CONFLICT OF INTEREST STATEMENT

The authors declare no conflicts of interest.
